# Assessment of current clinical practice throughout the UK for the diagnosis and management of monoclonal gammopathy of renal significance

**DOI:** 10.1002/jha2.658

**Published:** 2023-03-21

**Authors:** Satarupa Choudhuri, Francesco Rainone

**Affiliations:** ^1^ Department of Haematology Northern Care Alliance Manchester UK; ^2^ Department of Nephrology Northern Care Alliance Manchester UK

**Keywords:** MGRS, monoclonal gammopathy of renal significance

## Abstract

Since the inception of the term monoclonal gammopathy of renal significance (MGRS) in 2012 by the International Kidney and Monoclonal Gammopathy Research Group, there have been no consensus guidelines specifically pertaining to the UK regarding to patient management. We aimed to identify both regional and cross‐discipline variation in current clinical practice, to provide insight and rationale for a potential standardised pathway in the future. A national survey of 88 consultants from the disciplines of haematology and nephrology was conducted between June 2020 and July 2021. Agreement was evident for aspects of the diagnostic pathway, including presenting features likely to raise suspicion of MGRS and the most pertinent confounding factors to consider before renal biopsy. However, significant variability was identified in both the cohort of diagnostic tests used, as well as urinary work‐up for patients with suspected MGRS. Treatment and monitoring frequency was also an aspect of management identified as variable. Despite differences in clinical practice across the UK, MGRS diagnosis was widely regarded to be the joint responsibility of both disciplines. The results provide an indication of inter‐regional and interdisciplinary differences in practice, highlighting the need for improved awareness and standardised protocol for management of MGRS that applies to the UK population.

## 1 INTRODUCTION

The International Kidney and Monoclonal Gammopathy Research Group (IKMG) first proposed the term monoclonal gammopathy of renal significance (MGRS) to distinguish patients with renal diseases related to an underlying haematological disorder requiring treatment from those with the more benign entity, monoclonal gammopathy of undetermined significance [1]. MGRS encompasses a spectrum of haematological conditions that produce a monoclonal immunoglobulin (MIg) associated with kidney injury that otherwise do not meet the criteria for overt multiple myeloma or other lymphoproliferative neoplasms [2]. The guidelines were updated to include any B cell or plasma cell disorder with kidney lesions directly attributable to the MIg, which otherwise do not meet current haematologic criteria for treatment [3].

MGRS‐associated lesions may also occur in the absence of monoclonal immunoglobulin deposits via indirect mechanisms, and the definition of MGRS has expanded to include both C3 glomerulopathy and thrombotic microangiopathy, which may be secondary to a monoclonal gammopathy [3].

The risk of decline in renal function and the potential for irreversible organ damage to occur necessitates diagnosis to be expedited [4, 5, 6]. However, this is complicated by the non‐specificity associated with many MGRS symptoms [1]. Renal biopsy remains an essential requirement for a definitive diagnosis, but carries associated risks [3]. Therefore, establishment of a diagnosis of MGRS requires cross‐discipline awareness and cooperation between nephrology and haematology.

Chemotherapy targeted to the clone responsible for monoclonal protein secretion remains the only therapeutic option [1, 7, 8]. Once a diagnosis is established, treatment should be commenced urgently to preserve renal function [9, 10, 11, 12, 13]. However, the monitoring criteria and frequency of response assessment in these patients currently vary in the absence of standardised pathways.

We present the results from a national survey concerning the clinical practice in MGRS. Its purpose is to establish current practice and diagnostic challenges in MGRS across the UK and to identify the areas of concordance or disparity at both a regional level and across the relevant specialities.

## 2 MATERIALS AND METHODS

A questionnaire was developed on the AllCounted platform (AllCounted, Inc., Rockville, Maryland, USA https://www.allcounted.com). The survey was conducted between 24th June 2020 and 7th July 2021 and circulated by email and social network sites to consultants working within the specialities of haematology and nephrology. Participants were required to complete at least 60% of the questions for the response to be included within the results. Answers left blank by respondents were omitted from analyses.

Coding of free text responses was performed using QDA Miner Lite (Provalis Research, Montreal, Canada). Applying qualitative research methodology, responses were converted into keywords in the context of the questions posed [14]. The number of designations recorded for a specific parameter was summated, and these values were used to assign a rank order to determine the relative frequency.

## 3 RESULTS

### 3.1 Survey population

In total, 113 individuals commenced the survey, and 88 recorded a response suitable for analysis. Data were collected across the UK and participants grouped into larger geographic regions for subsequent analysis (Table 1).

**TABLE 1:** Demographics of monoclonal gammopathy of renal significance (MGRS) survey participants.

	Number of respondents			
UK Region	Haematology	Nephrology	Total	Total per region
Scotland	2	2	4	32 (North)
North East	2	0	2	
North West	6	18	24	
Northern Ireland	1	1	2	
Yorkshire and Humber	5	6	11	14 (East)
East Midlands	0	2	2	
East of England	0	1	1	
Wales	4	1	5	19 (West)
West Midlands	2	3	5	
South West	7	2	9	
South East	3	2	5	22 (South)
London	8	9	17	
Unknown^a^	1	0	1	
Total	41	47	88	

^a^ One individual completed all other survey questions but did not provide their region. They are included in comparative analysis between nephrology and haematology but are omitted from regional analysis.

Following the establishment of MGRS as a distinct clinical entity, awareness of the condition has increased. Participants from each specialty were asked how frequently they saw MGRS patients (new and follow‐up) in their clinic. Of the two disciplines, nephrologists reported that they were more likely to see MGRS patients each month (>4 per month: 12.8% vs. 7.3%; <4 per month: 51.1% vs. 51.2%; rarely: 36.2% vs. 41.5%).

### 3.2 Presenting features

**FIGURE 1:**
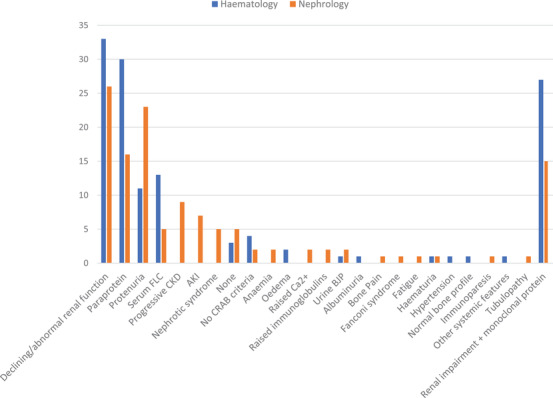
Features considered to be most predictive of monoclonal gammopathy of renal significance (MGRS). Absolute values for the number of responses received for a given presenting feature are provided. Haematology (*n* = 37), nephrology (*n* = 46).

Distinguishing MGRS from other monoclonal gammopathies and renal diseases can be challenging due to non‐specificity of presenting symptoms. Clinicians were asked to identify which features they considered to be most indicative of a potential MGRS diagnosis (Figure 1). Free text responses were coded to enable quantitative analysis. A composite category—renal impairment and paraprotein—was created for responses, which clearly indicated that the coincidence of both these features were important (42/83 ‐ 52.6%).

Haematologists and nephrologists identified declining renal function as the feature most indicative of MGRS (89.2% and 56.5%, respectively). For haematology, this was followed by the presence of a paraprotein of any type (83.8%), with monoclonal free light chains identified specifically by 35.1% of participants.

In contrast, nephrologists made specific reference to proteinuria in 50.0% of responses received. Monoclonal protein involvement was indicated less frequently by nephrologists than haematologists (paraprotein 34.8% vs. 83.8%, serum free light chain (FLC) 10.9% vs. 35.1%, respectively). Notably, 73.0% of haematologists indicated that concurrent symptoms of both renal impairment and the presence of a monoclonal protein were indicative of MGRS, compared to 32.6% of nephrologists (Table S1).

To investigate geographic variability, the features considered to be most predictive of MGRS were ranked according to the frequency of responses and subdivided according to the participant's regions (Table S2).

Despite slight differences in rank order, declining or abnormal renal function, proteinuria and evidence of monoclonal protein production were the three most frequently identified MGRS features, regardless of region. Deteriorating renal function: South – 59.1%, North – 65.6%, West – 84.2%, East – 64.3%. Paraprotein: South – 63.6%, North – 50.0%, West – 52.6%, East – 42.9%. Proteinuria: South – 45.5%, North – 40.6%, West – 15.8%, East – 57.1%.

Interestingly, based upon the number of responses, progressive CKD was ranked the 4th most predictive MGRS feature by clinicians from the North (15.6%) and East (21.4%) but ranked 9th in the South (4.5%) and was not mentioned by any clinician from the West (0.0%).

### 3.3 Diagnostic tests

**FIGURE 2:**
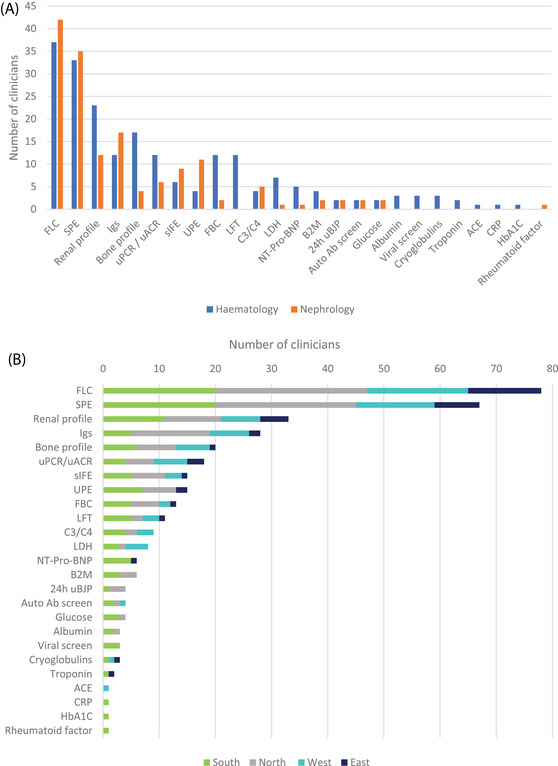
Tests requested to inform monoclonal gammopathy of renal significance (MGRS) diagnosis (A) by discipline, (B) by region (haematology, *n* = 41, nephrology, *n* = 47).

To gauge clinical practice, respondents were asked to provide an indication of which tests they would request to make a diagnosis of MGRS. Twenty‐five tests were identified by the participants (Figure 2A).

The most frequently requested tests to identify MGRS from both specialties were serum free light chain assay (sFLC) (86.0% haematology, 89.4% nephrology) and serum protein electrophoresis (SPE) (76.7% haematology, 74.5% nephrology). Renal and bone profiles were more frequently utilised by haematology (56.1% vs. 25.5% and 41.5% vs. 8.5%, respectively). In contrast, immunoglobulin tests and urine protein electrophoresis were more frequently requested by nephrology (Igs; 36.2% vs. 29.3%, urinary protein and albumin (UPE); 23.4% vs. 9.8%).

The frequency of requests for FBCs, LFTs and LDH was also noticeably greater for haematology than nephrology (full blood count (FBC); 29.3% vs. 4.3%, liver function test (LFT); 29.3% vs. 0.0%, LDH; 17.1% vs. 2.1%). Of note, haematologists were more likely than nephrologists to request NT‐proBNP (12.2% vs. 2.1%).

We also wanted to determine if there were regional differences in tests requested (Figure 2B). Serum free light chain testing and SPE were the most frequently ordered tests in all regions of the UK. Ten of 25 tests were ordered at least once by a clinician from each region.

Significant differences were identified in the tests requested across regions by both specialities. Clinicians from the East region requested SPE less frequently (57.1% vs. UK average – 75.0%), whereas respondents from the North were more likely to request immunoglobulin testing (48.8% vs. UK average – 29.4%). UPE requesting was highly variable, being used most frequently by clinicians in the Southern region (31.8% vs. UK average – 17.0%). In contrast, no respondents from the West reported that they would request UPE. NT‐ProBNP was ordered by 22.7% of clinicians from the South region and 7.1% from the East but was not requested by any clinician from the other regions.

Participants were asked which tests they would request, in order to understand their current practice (Table 2).

**TABLE 2:** Use of urine tests by discipline and region.

	Frequency (Percentage of total)						
Discipline	ACR only	PCR only	Glucose only	ACR + PCR	ACR + Glucose	PCR + Glucose	ACR + PCR + Glucose
Haematology (*n* = 38)	7 (18.4%)	12 (31.4%)	0	15 (39.5%)	2 (5.3%)	0	2 (5.3%)
Nephrology (*n* = 46)	2 (4.3%)	20 (43.5%)	0	17 (37%)	1 (2.2%)	4 (8.7%)	2 (4.3%)

Region							
North (*n* = 32)	6 (18.8%)	13 (40.6%)	0	10 (31.3%)	1 (3.1%)	2 (6.3%)	0
East (*n* = 14)	0	4 (28.6%)	0	8 (57.1%)	1 (7.1%)	1 (7.1%)	0
West (*n* = 18)	1 (5.6%)	8 (44.4%)	0	8 (44.4%)	0	0	1 (5.6%)
South (*n* = 20)	2 (10.0%)	7 (35.0%)	0	6 (30.0%)	1 (5.0%)	1 (5.0%)	3 (15.0%)

Abbreviations: ACR, Albumin to creatinine ratio; PCR, Protein creatinine ratio.

Protein/creatinine ratio and protein/creatinine ratio and albumin/creatinine ratio were the most frequently used urine tests across the two disciplines. Protein/creatinine ratio was favoured by nephrologists (43.5%), whereas a combination of both protein/creatinine ratio and albumin/creatinine ratio was preferred by haematologists (39.5%).

The most popular tests in the East region were protein/creatinine ratio and albumin/creatinine ratio (57.1%), whereas protein/creatinine ratio was used most frequently in the other regions. The South had the greatest proportion of clinicians requesting all three tests.

### 3.4 Monoclonal protein involvement

Identification of the causative monoclonal immunoglobulin is important for MGRS diagnosis and prognostication. We sought to understand which combinations of tests were routinely used to determine monoclonal protein involvement. Serum free light chain investigation and SPE were most frequently used to detect monoclonal proteins with 82/88 (93.2%) and 81/88 (92.0%) of participants indicating they used each test, respectively.

Twenty‐four different combinations of tests were identified from respondents. Of these, six were unique to nephrology and seven unique to haematology, indicating differences between the two specialties (Table S3). The testing combinations were ranked according to frequency of use and the five cohorts of tests that were most frequently requested are displayed in Table 3.

**TABLE 3:** Diagnostic tests to determine monoclonal protein involvement.

	Combination of tests							Frequency (per cent of total)		
Rank	SPE	sIFE	UPE	uIFE	sFLC	Igs	24 h uBJP	Haematology	Nephrology	Total
1	✓	✓	✓	✓	✓	✓		10 (24.4%)	9 (19.1%)	19
= 2	✓	✓			✓	✓		9 (22.0%)	3 (6.4%)	12
=2	✓				✓	✓		5 (12.2%)	7 (14.9%)	12
4	✓		✓		✓	✓		1 (2.4%)	7 (14.9%)	8
5	✓				✓			3 (7.3%)	4 (8.5%)	7

The five most frequently used cohorts of tests were assigned a number that corresponded to their popularity amongst the surveyed clinicians. Regional differences were then assessed according to the frequency of use of these different tests (Table 4). Clinicians in the North, South and East regions favoured testing cohort 1 (SPE, sIFE, UPE, uIFE, sFLC and Igs), whereas 8/19 (42.1%) from the West preferred testing cohort 2 (SPE, sIFE, sFLC and Igs). These differences may be reflective of local/regional guidelines.

**TABLE 4:** Frequency of the most popular cohorts of tests used to determine monoclonal protein involvement according to region.

	UK region frequency (per cent)			
Test cohort	South	North	West	East
1 – SPE, sIFE, UPE, uIFE, sFLC, Igs	5 (22.7%)	6 (18.2%)	2 (10.5%)	5 (35.7%)
2 – SPE, sIFE, sFLC, Igs	1 (4.5%)	1 (3.0%)	8 (42.1%)	1 (7.1%)
3 – SPE, sFLC, Igs	3 (13.6%)	4 (12.1%)	2 (10.5%)	2 (14.3%)
4 – SPE, UPE, sFLC, Igs	3 (13.6%)	2 (6.1%)	2 (10.5%)	1 (7.1%)
5 – SPE, sFLC	3 (13.6%)	1 (3.0%)	2 (10.5%)	1 (7.1%)

Differential filtration of free light chains by the kidneys is affected by renal impairment and, therefore, should be accounted for when interpreting slightly abnormal FLC ratios in these patients. We asked respondents whether they used a renal reference range when interpreting sFLC results, such as that proposed by Hutchison and colleagues [15]. The greatest proportion of clinicians using a renal reference range was 81.8% in the South (nephrology, 81.8%; haematology, 81.8%), followed by 72.9% in the North (nephrology, 85.7%; haematology, 60.0%), 60.7% in the West (nephrology, 50.0%; haematology, 71.4%) and 57.8% of participants from the East (nephrology, 55.6%; haematology, 60.0%).

### 3.5 Renal biopsy

Participants were asked which factors were most pertinent when considering a renal biopsy (Table 5). Risk of bleeding was considered by both specialities to be the most important, with 94.6% of haematologists and 81.4% of nephrologists indicating this to be of concern. This was followed by age and severe renal impairment (age 56.8% and 39.5%, severe renal impairment 48.6% and 39.5%, haematologist and nephrologists, respectively). In each instance, a greater proportion of haematologists considered these features to be confounding. Additional factors identified by participants included small/single kidney (6.25%), frailty (5%) and whether the result of the biopsy was likely to change treatment (3.8%).

**TABLE 5:** Most pertinent confounding factors before considering kidney biopsy.

	Age	Bleeding risk	Diabetes	Severe renal impairment
Haematology	21 (56.8%)	35 (94.6%)	9 (24.3%)	18 (48.6%)
Nephrology	17 (39.5%)	35 (81.4%)	4 (9.3%)	17 (39.5%)
Total	38 (47.5%)	70 (87.5%)	13 (16.3%)	35 (43.8%)

Clinicians were asked which speciality they believe has the responsibility for diagnosing MGRS. A proportion of participants 12/88 (13.6%) felt that this duty lies with nephrology and 2/88 (2.3%) with haematology, but there was strong consensus overall that the responsibility for the diagnosis of these patients should be jointly shared by both specialities 74/88 (84.1%).

### 3.6 Response and monitoring

We asked which parameters were considered most critical in determining the treatment administered in MGRS. Suppression of the monoclone was considered the most important factor by 35/79 (44.3%) respondents. Improvement in eGFR and suppression of the monoclone were deemed to be of equal importance for 23/79 (29.1%) clinicians, and 21/79 (26.6%) identified improvement of eGFR as most critical.

Clinicians were asked which treatment modalities they use. Note that 89.2% haematologists and 51.2% nephrologists reported that they use clone‐directed therapy only. 10.8% haematologists and 30.2% nephrologists indicated they would use both clone‐directed and immunosuppressive modalities, and 4.7% nephrologists would use immunosuppressive therapy only. Note that 11.5% nephrologists indicated that choice of treatment would be led by haematology.

**FIGURE 3:**
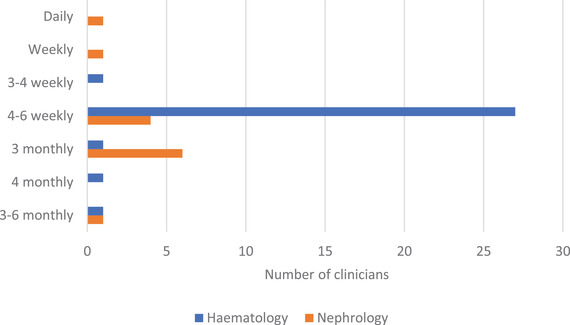
Reported frequency of monitoring for haematological response, according to speciality.

Virtually all respondents (98.8%) reported that they monitor renal response and 84.1% monitor haematological response. We sought to understand which criteria are being used to assess the response of MGRS patients to treatment and the frequency of monitoring. The frequency of monitoring reported by both specialities is displayed in Figure 3.

Disparity was noted in the frequency of monitoring, with a higher proportion of haematologists following up patients on a 4–6 weekly basis, with ad hoc nephrology input (87.1% haematology, 30.8% nephrology). Responses to the open‐ended question revealed that it was common (48.6%) for nephrologists to refer patients to haematology for monitoring.

The most frequent standard used by haematologists was the International Myeloma Working Group (IMWG) myeloma response criteria (20/38, 52.6%), followed by the amyloidosis (AL) amyloidosis criteria (13/38, 34.2%), and several individuals reported that they used both (5/38, 13.2%). In contrast, a greater proportion of nephrologists use the AL amyloidosis criteria (14/42, 33.3%), as opposed to the IMWG (9/42, 21.4%), with 2/42 (4.8%) using both.

## 4 DISCUSSION

The results of this survey provide insight into current clinical practice concerning MGRS across the disciplines of both haematology and nephrology as well as regional differences in MGRS management. These results represent, to our knowledge, the first national survey on current clinical practice for MGRS.

Of the two disciplines, nephrologists were more likely to see MGRS patients, which is perhaps due to the renal abnormalities provoked by the disease. Interestingly, there were no differences in the reported frequency of referrals from haematology to nephrology and vice versa. MGRS diagnosis can prove challenging, so participants were asked to indicate the presenting features that they most associate with these conditions, to provide an indication of symptom awareness and reflect their experience. In the absence of national guidelines, we surmised that there might be regional differences in the features deemed most predictive of a diagnosis.

Consensus was evident amongst clinicians that declining renal function in association with an accompanying paraprotein, particularly monoclonal light chains, is the most indicative feature. This awareness was particularly high amongst haematologists (73.0% vs. 32.6% nephrologists). Interestingly, knowledge of the association between both features was significantly higher amongst consultants from London and the South East, possibly owing to increased awareness linked to their proximity to the National Amyloidosis Centre.

A greater range of clinical features were identified by nephrologists, with 19 different features reported, compared to 13 listed by haematologists. Whilst haematologists perceived declining renal function as most indicative of MGRS diagnosis, nephrologists deemed new proteinuria and progressive chronic kidney disease (CKD) or acute kidney injury (AKI) most indicative of the diagnosis. On the other hand, monoclonal protein involvement was indicated less frequently by nephrologists (paraprotein 34.8% vs. 57.1%, serum FLC 10.9% vs. 35.1%; nephrology vs. haematology).

Haematologists focused on the identification of a monoclonal protein and exclusion of other malignancies. A lack of other myeloma‐defining events was identified as important to rule out a diagnosis of multiple myeloma. This difference in approach to testing between specialties reflects a lack of standardised diagnostic protocol and testing priorities based on specialty perspectives.

Whilst SPE and sFLC were the most frequently requested tests, variation between the specialities regarding the less frequently ordered tests was noted. For example, autoimmune screening was mentioned by few clinicians to rule out glomerulonephritis. Of the testing cohorts identified, 13 were unique to one discipline or the other, indicating considerable variability in requesting. This reflects the differences amongst clinicians in perception and interpretation of MGRS as a disease and the investigations performed for diagnosis and prognostication.

MGRS guidelines from the IKMG and AL amyloidosis guidelines from the British Society for Haematology recommend urine testing as part of the work‐up for patients with suspected MGRS [7, 16]. However, there seemed to be a lack of uniformity in specific urinary tests performed across the regions, with significant variation noted in uACR, uPCR, dual tests and glucose. Obtaining uACR and uPCR simultaneously is important in proteinuric patients as this informs whether the aetiology is within the glomerulus—resulting in high uACR, as in amyloidosis—or if it is linked FLC overflow (therefore normal uACR but high uPCR). Glucose testing provides information regarding possible diabetic nephropathy. These results are further confounded by regional variation in the availability and funding of specific tests, again highlighting the need for standardisation.

Declining renal function should be accounted for whilst interpreting mildly abnormal FLC ratios [3, 17 ^–^ 19]. The variability of FLC ratio depends on the assay used [20, 21]. Since Freelite is the most widely used FLC assay in the UK, we asked respondents whether they used a renal reference range when interpreting results. There was no difference identified in the overall utilisation of a renal reference range according to speciality. However, there were regional differences, with the greatest proportion of clinicians utilising a renal reference range in the South (81.8%).

Renal biopsy is recommended by the IKMG for a definitive diagnosis of MGRS [2, 3]. Whilst the procedure carries inherent‐associated risks, rates of haemorrhagic complications have been found to be similar in patients with and without MGRS [22, 23]. To assess perception about renal biopsy, participants were asked about confounding factors. The responses were concordant. Risk of bleeding was identified as the most pertinent factor, followed by age and severe renal impairment. Notably, a higher proportion of haematologists raised concerns (Table 6), which may be due to a lack of practical experience, since renal biopsy is a procedure performed exclusively by nephrologists. This disparity in perception should be addressed by raising awareness of the condition so that it does not preclude the referral of a patient for renal biopsy, where appropriate.

**TABLE 6:** Most pertinent confounding factors before considering kidney biopsy.

	Age	Bleeding risk	Diabetes	Severe renal impairment
Haematology	21 (56.8%)	35 (94.6%)	9 (24.3%)	18 (48.6%)
Nephrology	17 (39.5%)	35 (81.4%)	4 (9.3%)	17 (39.5%)
Total	38 (47.5%)	70 (87.5%)	13 (16.3%)	35 (43.8%)

There was significant variability noted in the frequency of follow‐up appointments scheduled by the two specialities. This highlights a potential concern of inadequate monitoring, identification of adverse events of treatment delivered and early relapse.

There is strong consensus and appetite from clinicians of both disciplines to share responsibility for patients with MGRS. A joint approach provides expertise on clone directed chemotherapy as well as appropriate management of renal issues. This raises an issue regarding potential establishment of joint clinics and regional multidisciplinary team discussions for the cross‐speciality management of these patients and improved outcomes.

In conclusion, this large survey generates important real‐world information, albeit with limitations stemming from variability in regional responses to the survey and differing availability of tests for each centre. Whilst aspects of regional and interdisciplinary variation in the management of MGRS patients were expected, this research represents, to our knowledge, the first substantiated evidence of this assumption. The data presented here underscore the need to establish dual discipline services for effective patient management.

## CONFLICT OF INTEREST STATEMENT

The authors declare that there is no conflict of interest that could be perceived as prejudicing the impartiality of the research reported.

## Supporting information

Supporting Information

## References

[1] Leung N , Bridoux F , Hutchison C , Nasr S , Cockwell P , Fermand J , et al. Monoclonal gammopathy of renal significance: when MGUS is no longer undetermined or insignificant. Blood. 2012;120(22):4292–5. 23047823 10.1182/blood-2012-07-445304

[2] Bridoux F , Leung N , Hutchison C , Touchard G , Sethi S , Fermand J , et al. Diagnosis of monoclonal gammopathy of renal significance. Kidney Int. 2015;87(4):698–711. 25607108 10.1038/ki.2014.408

[3] Leung N , Bridoux F , Batuman V , Chaidos A , Cockwell P , D'Agati V , et al. The evaluation of monoclonal gammopathy of renal significance: a consensus report of the International Kidney and Monoclonal Gammopathy Research Group. Nat Rev Nephrol. 2018;15(1):45–59. 10.1038/s41581-018-0077-4 PMC7136169 30510265

[4] Pinney J , Lachmann H , Bansi L , Wechalekar A , Gilbertson J , Rowczenio D , et al. Outcome in renal AL amyloidosis after chemotherapy. J Clin Oncol. 2011;29(6):674–81. 21220614 10.1200/JCO.2010.30.5235

[5] Nasr S , Valeri A , Cornell L , Fidler M , Sethi S , D'Agati V , et al. Renal monoclonal immunoglobulin deposition disease: a report of 64 patients from a single institution. Clin J Am Soc Nephrol. 2011;7(2):231–9. 22156754 10.2215/CJN.08640811

[6] Kourelis T , Nasr S , Dispenzieri A , Kumar S , Gertz M , Fervenza F , et al. Outcomes of patients with renal monoclonal immunoglobulin deposition disease. Am J Hematol. 2016;91(11):1123–8. 27501122 10.1002/ajh.24528

[7] Fermand J , Bridoux F , Kyle R , Kastritis E , Weiss B , Cook M , et al. How I treat monoclonal gammopathy of renal significance (MGRS). Blood. 2013;122(22):3583–90. 24108460 10.1182/blood-2013-05-495929

[8] Dimopoulos M , Sonneveld P , Leung N , Merlini G , Ludwig H , Kastritis E , et al. International Myeloma Working Group recommendations for the diagnosis and management of myeloma‐related renal impairment. J Clin Oncol. 2016;34(13):1544–57. 26976420 10.1200/JCO.2015.65.0044

[9] Hogan J , Weiss B . Bridging the divide: an onco‐nephrologic approach to the monoclonal gammopathies of renal significance. Clin J Am Soc Nephrol. 2016;11(9):1681–91. 27416775 10.2215/CJN.03160316 PMC5012477

[10] Rosner M , Edeani A , Yanagita M , Glezerman I , Leung N . Paraprotein–related kidney disease: diagnosing and treating monoclonal gammopathy of renal significance. Clin J Am Soc Nephrol. 2016;11(12):2280–7. 27526705 10.2215/CJN.02920316 PMC5142062

[11] Chauvet S , Frémeaux‐Bacchi V , Petitprez F , Karras A , Daniel L , Burtey S , et al. Treatment of B‐cell disorder improves renal outcome of patients with monoclonal gammopathy–associated C3 glomerulopathy. Blood. 2017;129(11):1437–47. 28069603 10.1182/blood-2016-08-737163

[12] Gumber R , Cohen J , Palmer M , Kobrin S , Vogl D , Wasserstein A , et al. A clone‐directed approach may improve diagnosis and treatment of proliferative glomerulonephritis with monoclonal immunoglobulin deposits. Kidney Int. 2018;94(1):199–205. 29759418 10.1016/j.kint.2018.02.020

[13] Jain A , Haynes R , Kothari J , Khera A , Soares M , Ramasamy K . Pathophysiology and management of monoclonal gammopathy of renal significance. Blood Adv. 2019;3(15):2409–23. 31409583 10.1182/bloodadvances.2019031914 PMC6693003

[14] Kuckartz U . Qualitative text analysis. London, UK: SAGE Publications; 2014.

[15] Hutchison C , Basnayake K , Cockwell P . Serum free light chain assessment in monoclonal gammopathy and kidney disease. Nat Rev Nephrol. 2009;5(11):621–8. 19786994 10.1038/nrneph.2009.151

[16] Gillmore J , Wechalekar A , Bird J , Cavenagh J , Hawkins S , Kazmi M , et al. Guidelines on the diagnosis and investigation of AL amyloidosis. Br J Haematol. 2014;168(2):207–18. 25312307 10.1111/bjh.13156

[17] Hutchison C , Harding S , Hewins P , Mead G , Townsend J , Bradwell A , et al. Quantitative assessment of serum and urinary polyclonal free light chains in patients with chronic kidney disease. Clin J Am Soc Nephrol. 2008;3(6):1684–90. 18945993 10.2215/CJN.02290508 PMC2572283

[18] Dispenzieri A , Kyle R , Merlini G , Miguel J , Ludwig H , Hajek R , et al. International Myeloma Working Group guidelines for serum‐free light chain analysis in multiple myeloma and related disorders. Leukemia. 2008;23(2):215–24. 19020545 10.1038/leu.2008.307

[19] Leung N , Barnidge D , Hutchison C . Laboratory testing in monoclonal gammopathy of renal significance (MGRS). Clin Chem Lab Med. 2016;54(6):929‐37. 10.1515/cclm-2015-0994 27107835

[20] Kennard A , Hawley C , Tate J , Klingberg S , Pretorius C , Hutchison C , et al. Comparison of Freelite™ and N Latex serum free light chain assays in subjects with end stage kidney disease on haemodialysis. Clin Chem Lab Med. 2016;54(6):1045–52. 10.1515/cclm-2015-0799 26684350

[21] Sprangers B , Claes K , Evenepoel P , Kuypers D , Poesen K , Delforge M , et al. Comparison of 2 serum‐free light‐chain assays in CKD patients. Kidney Int Rep. 2020;5(5):627–31. 32405584 10.1016/j.ekir.2020.01.019 PMC7210599

[22] Fish R , Pinney J , Jain P , Addison C , Jones C , Jayawardene S , et al. The incidence of major hemorrhagic complications after renal biopsies in patients with monoclonal gammopathies. Clin J Am Soc Nephrol. 2010;5(11):1977–80. 20651154 10.2215/CJN.00650110 PMC3001781

[23] Bakdash K , Schramm K , Annam A , Brown M , Kondo K , Lindquist J . Complications of percutaneous renal biopsy. Semin Interve Radiol. 2019;36(02):097–103. 10.1055/s-0039-1688422 PMC6531025 31123379

