# Supplementary material for: Assessment of current clinical practice throughout the UK for the diagnosis and management of monoclonal gammopathy of renal significance
Source: EJHaem. 2023 Mar 21;4(2):410–8. doi: 10.1002/jha2.658 (PMC10188446; doi:10.1002/jha2.658)
Supplement: Supplementary file 1 — Supporting Information [file JHA2-4-410-s001.docx]

**Supplemental Materials**

**Supplemental Table 1** - Features considered to be most predictive of MGRS according to discipline (Haematology n=37, Nephrology n=46). The frequency of responses received for a given presenting feature are provided. An additional category - renal impairment + monoclonal protein – includes responses indicating these features should be coincident.

|  | Haematology | | Nephrology | | |
| --- | --- | --- | --- | --- | --- |
| Presenting Feature | **Frequency** | **Percentage** | **Frequency** | | **Percentage** |
| Declining/abnormal renal function | 33 | 89.2 | 26 | 56.5 | |
| Paraprotein | 30 | 81.1 | 16 | 34.8 | |
| Proteinuria | 11 | 29.7 | 23 | 50.0 | |
| Serum FLC | 13 | 35.1 | 5 | 10.9 | |
| Progressive CKD | 0 | 0.0 | 9 | 19.6 | |
| AKI | 0 | 0.0 | 7 | 15.2 | |
| Nephrotic syndrome | 0 | 0.0 | 5 | 10.9 | |
| None | 3 | 8.1 | 5 | 10.9 | |
| No CRAB criteria | 4 | 10.8 | 2 | 4.3 | |
| Anaemia | 0 | 0.0 | 2 | 4.3 | |
| Oedema | 2 | 5.4 | 0 | 0.0 | |
| Raised Ca^2+^ | 0 | 0.0 | 2 | 4.3 | |
| Raised immunoglobulins | 0 | 0.0 | 2 | 4.3 | |
| Urine BJP | 1 | 2.7 | 2 | 4.3 | |
| Albuminuria | 1 | 2.7 | 0 | 0.0 | |
| Bone Pain | 0 | 0.0 | 1 | 2.2 | |
| Fanconi syndrome | 0 | 0.0 | 1 | 2.2 | |
| Fatigue | 0 | 0.0 | 1 | 2.2 | |
| Haematuria | 1 | 2.7 | 1 | 2.2 | |
| Hypertension | 1 | 2.7 | 0 | 0.0 | |
| Normal bone profile | 1 | 2.7 | 0 | 0.0 | |
| Immunoparesis | 0 | 0.0 | 1 | 2.2 | |
| Other systemic features | 1 | 2.7 | 0 | 0.0 | |
| Tubulopathy | 0 | 0.0 | 1 | 2.2 | |
| Renal impairment + monoclonal protein | 27 | 73.0 | 15 | 32.6 | |

**Supplemental Table 2**. Features considered to be most predictive of MGRS according to region. Presenting features identified by nephrologists (n=46) and haematologists (n=37) to be predictive of MGRS are divided according to their respective geographic regions. Features were then ranked according to the frequency of responses by region, to determine relative importance.

|  | UK Region | | | | | | | |
| --- | --- | --- | --- | --- | --- | --- | --- | --- |
|  | **South (n=22)** | | **North (n=32)** | | **West (n=19)** | | **East (n=14)** | |
| Presenting Feature | Number | Rank | Number | Rank | Number | Rank | Number | Rank |
| Paraprotein | 14 | 1 | 16 | 2 | 10 | 2 | 6 | 3 |
| Declining/abnormal renal function | 13 | 2 | 21 | 1 | 16 | 1 | 9 | 1 |
| Proteinuria | 10 | 3 | 13 | 3 | 3 | =3 | 8 | 2 |
| Serum FLC | 7 | 4 | 5 | =4 | 3 | =3 | 3 | =4 |
| None | 4 | 5 | 2 | =7 | 2 | =5 | 0 | - |
| AKI | 3 | 6 | 3 | 6 | 0 | - | 1 | =6 |
| Nephrotic syndrome | 2 | =7 | 2 | =7 | 0 | - | 1 | =6 |
| Haematuria | 2 | =7 | 0 | - | 0 | - | 0 | - |
| Progressive CKD | 1 | =9 | 5 | =4 | 0 | - | 3 | =4 |
| No CRAB criteria | 1 | =9 | 2 | =7 | 2 | =5 | 1 | =6 |
| Raised Ca^2+^ | 1 | =9 | 1 | =11 | 0 | - | 0 | - |
| Raised immunoglobulins | 1 | =9 | 1 | =11 | 0 | - | 0 | - |
| Bone Pain | 1 | =9 | 0 | - | 0 | - | 0 | - |
| Immunoparesis | 1 | =9 | 0 | - | 0 | - | 0 | - |
| Normal bone profile | 1 | =9 | 0 | - | 0 | - | 0 | - |
| Other systemic features | 1 | =9 | 0 | - | 0 | - | 0 | - |
| Tubulopathy | 1 | =9 | 0 | - | 0 | - | 0 | - |
| Anaemia | 0 | - | 2 | =7 | 0 | - | 0 | - |
| Oedema | 0 | - | 0 | - | 1 | =8 | 1 | =6 |
| Urine BJP | 0 | - | 1 | =11 | 2 | =5 | 0 | - |
| Hypertension | 0 | - | 0 | - | 1 | =8 | 0 | - |
| Fanconi syndrome | 0 | - | 1 | =11 | 0 | - | 0 | - |
| Fatigue | 0 | - | 1 | =11 | 0 | - | 0 | - |
| Albuminuria | 0 | - | 0 | - | 1 | =8 | 0 | - |

**Supplemental Table 3**. Diagnostic tests to determine monoclonal protein involvement (Haematology n=41, Nephrology n=47).

|  | Combination of tests | | | | | | | Frequency (percent of total) | |  |
| --- | --- | --- | --- | --- | --- | --- | --- | --- | --- | --- |
|  | **SPE** | **sIFE** | **UPE** | **uIFE** | **sFLC** | **Igs** | **24h uBJP** | **Haematology** | **Nephrology** | **Total** |
|  | ✓ | ✓ | ✓ | ✓ | ✓ | ✓ |  | 10 (24.4%) | 9 (19.1%) | 19 |
|  | ✓ | ✓ |  |  | ✓ | ✓ |  | 9 (22.0%) | 3 (6.4%) | 12 |
|  | ✓ |  |  |  | ✓ | ✓ |  | 5 (12.2%) | 7 (14.9%) | 12 |
|  | ✓ |  | ✓ |  | ✓ | ✓ |  | 1 (2.4%) | 7 (14.9%) | 8 |
|  | ✓ |  |  |  | ✓ |  |  | 3 (7.3%) | 4 (8.5%) | 7 |
|  | ✓ | ✓ | ✓ | ✓ | ✓ | ✓ | ✓ | 2 (4.9%) | 2 (4.3%) | 4 |
|  | ✓ | ✓ | ✓ |  | ✓ | ✓ | ✓ | 1 (2.4%) | 2 (4.3%) | 3 |
|  | ✓ | ✓ |  |  | ✓ |  |  | 1 (2.4%) | 1 (2.1%) | 2 |
|  | ✓ |  |  |  |  | ✓ |  | 1 (2.4%) | 1 (2.1%) | 2 |
|  | ✓ | ✓ | ✓ |  | ✓ | ✓ |  | 1 (2.4%) | 1 (2.1%) | 2 |
| Haematology only | ✓ |  | ✓ |  | ✓ |  | ✓ | 1 (2.4%) |  | 1 |
|  | ✓ | ✓ |  | ✓ | ✓ | ✓ |  | 1 (2.4%) |  | 1 |
|  | ✓ | ✓ |  |  | ✓ | ✓ | ✓ | 1 (2.4%) |  | 1 |
|  | ✓ |  | ✓ | ✓ | ✓ | ✓ |  | 1 (2.4%) |  | 1 |
|  | ✓ | ✓ | ✓ | ✓ | ✓ |  |  | 1 (2.4%) |  | 1 |
|  |  |  |  |  | ✓ | ✓ |  | 1 (2.4%) |  | 1 |
|  | ✓ | ✓ |  | ✓ | ✓ | ✓ | ✓ | 1 (2.4%) |  | 1 |
| Nephrology only |  |  |  |  | ✓ |  |  |  | 3 (6.4%) | 3 |
|  | ✓ |  |  | ✓ | ✓ | ✓ |  |  | 2 (4.3%) | 2 |
|  | ✓ |  | ✓ |  | ✓ |  |  |  | 2 (4.3%) | 2 |
|  | ✓ |  |  |  |  |  |  |  | 1 (2.1%) | 1 |
|  | ✓ |  |  | ✓ |  |  |  |  | 1 (2.1%) | 1 |
|  |  | ✓ |  |  | ✓ | ✓ |  |  | 1 (2.1%) | 1 |
| Total | | | | | | | | 41 | 47 | 88 |
